# COVID‐19 Disease Map, a computational knowledge repository of virus‐host interaction mechanisms

**DOI:** 10.15252/msb.202110851

**Published:** 2021-12-23

**Authors:** Marek Ostaszewski, Anna Niarakis, Alexander Mazein, Inna Kuperstein, Robert Phair, Aurelio Orta‐Resendiz, Vidisha Singh, Sara Sadat Aghamiri, Marcio Luis Acencio, Enrico Glaab, Andreas Ruepp, Gisela Fobo, Corinna Montrone, Barbara Brauner, Goar Frishman, Luis Cristóbal Monraz Gómez, Julia Somers, Matti Hoch, Shailendra Kumar Gupta, Julia Scheel, Hanna Borlinghaus, Tobias Czauderna, Falk Schreiber, Arnau Montagud, Miguel Ponce de Leon, Akira Funahashi, Yusuke Hiki, Noriko Hiroi, Takahiro G Yamada, Andreas Dräger, Alina Renz, Muhammad Naveez, Zsolt Bocskei, Francesco Messina, Daniela Börnigen, Liam Fergusson, Marta Conti, Marius Rameil, Vanessa Nakonecnij, Jakob Vanhoefer, Leonard Schmiester, Muying Wang, Emily E Ackerman, Jason E Shoemaker, Jeremy Zucker, Kristie Oxford, Jeremy Teuton, Ebru Kocakaya, Gökçe Yağmur Summak, Kristina Hanspers, Martina Kutmon, Susan Coort, Lars Eijssen, Friederike Ehrhart, D A B Rex, Denise Slenter, Marvin Martens, Nhung Pham, Robin Haw, Bijay Jassal, Lisa Matthews, Marija Orlic‐Milacic, Andrea Senff‐Ribeiro, Karen Rothfels, Veronica Shamovsky, Ralf Stephan, Cristoffer Sevilla, Thawfeek Varusai, Jean‐Marie Ravel, Rupsha Fraser, Vera Ortseifen, Silvia Marchesi, Piotr Gawron, Ewa Smula, Laurent Heirendt, Venkata Satagopam, Guanming Wu, Anders Riutta, Martin Golebiewski, Stuart Owen, Carole Goble, Xiaoming Hu, Rupert W Overall, Dieter Maier, Angela Bauch, Benjamin M Gyori, John A Bachman, Carlos Vega, Valentin Grouès, Miguel Vazquez, Pablo Porras, Luana Licata, Marta Iannuccelli, Francesca Sacco, Anastasia Nesterova, Anton Yuryev, Anita de Waard, Denes Turei, Augustin Luna, Ozgun Babur, Sylvain Soliman, Alberto Valdeolivas, Marina Esteban‐Medina, Maria Peña‐Chilet, Kinza Rian, Tomáš Helikar, Bhanwar Lal Puniya, Dezso Modos, Agatha Treveil, Marton Olbei, Bertrand De Meulder, Stephane Ballereau, Aurélien Dugourd, Aurélien Naldi, Vincent Noël, Laurence Calzone, Chris Sander, Emek Demir, Tamas Korcsmaros, Tom C Freeman, Franck Augé, Jacques S Beckmann, Jan Hasenauer, Olaf Wolkenhauer, Egon L Willighagen, Alexander R Pico, Chris T Evelo, Marc E Gillespie, Lincoln D Stein, Henning Hermjakob, Peter D'Eustachio, Julio Saez‐Rodriguez, Joaquin Dopazo, Alfonso Valencia, Hiroaki Kitano, Emmanuel Barillot, Charles Auffray, Rudi Balling, Reinhard Schneider, the COVID‐19 Disease Map Community

## Abstract

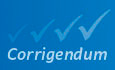

The authors requested to correct the spelling of Egon Willighagen and Andrea Senff‐Ribeiro's names, as well as the following affiliations: Charles Auffray to: “European Institute for Systems Biology and Medicine (EISBM), Vourles, France”; Noriko Hiori to: “Graduate School of Media and Governance, Keio Research Institute at SFC, Keio University, Kanagawa, Japan”; and Leonard Schmeister to: “Center for Mathematics, Chair of Mathematical Modeling of Biological Systems, Technische Universität München, Garching, Germany and Helmholtz Zentrum München – German Research Center for Environmental Health, Institute of Computational Biology, Neuherberg, Germany”.

